# Supplementary figures and images for: Mathematical Modeling of Fluconazole Resistance in the Ergosterol Pathway of Candida albicans
Source: mSystems. 2022 Nov 16;7(6):e00691-22. doi: 10.1128/msystems.00691-22 (PMC9765018; doi:10.1128/msystems.00691-22)

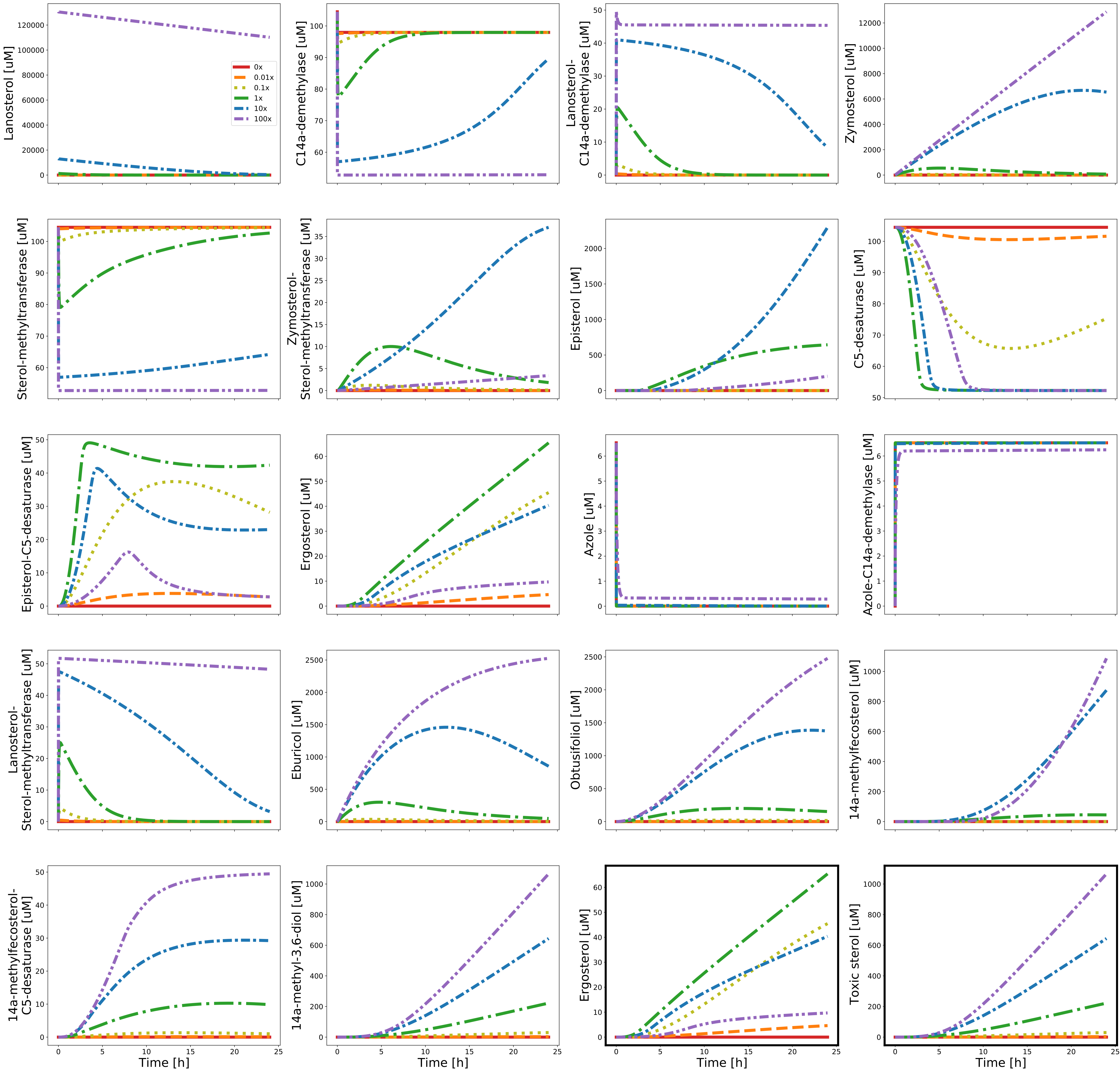

Supplement: FIG S2 [file msystems.00691-22-s0003.pdf]

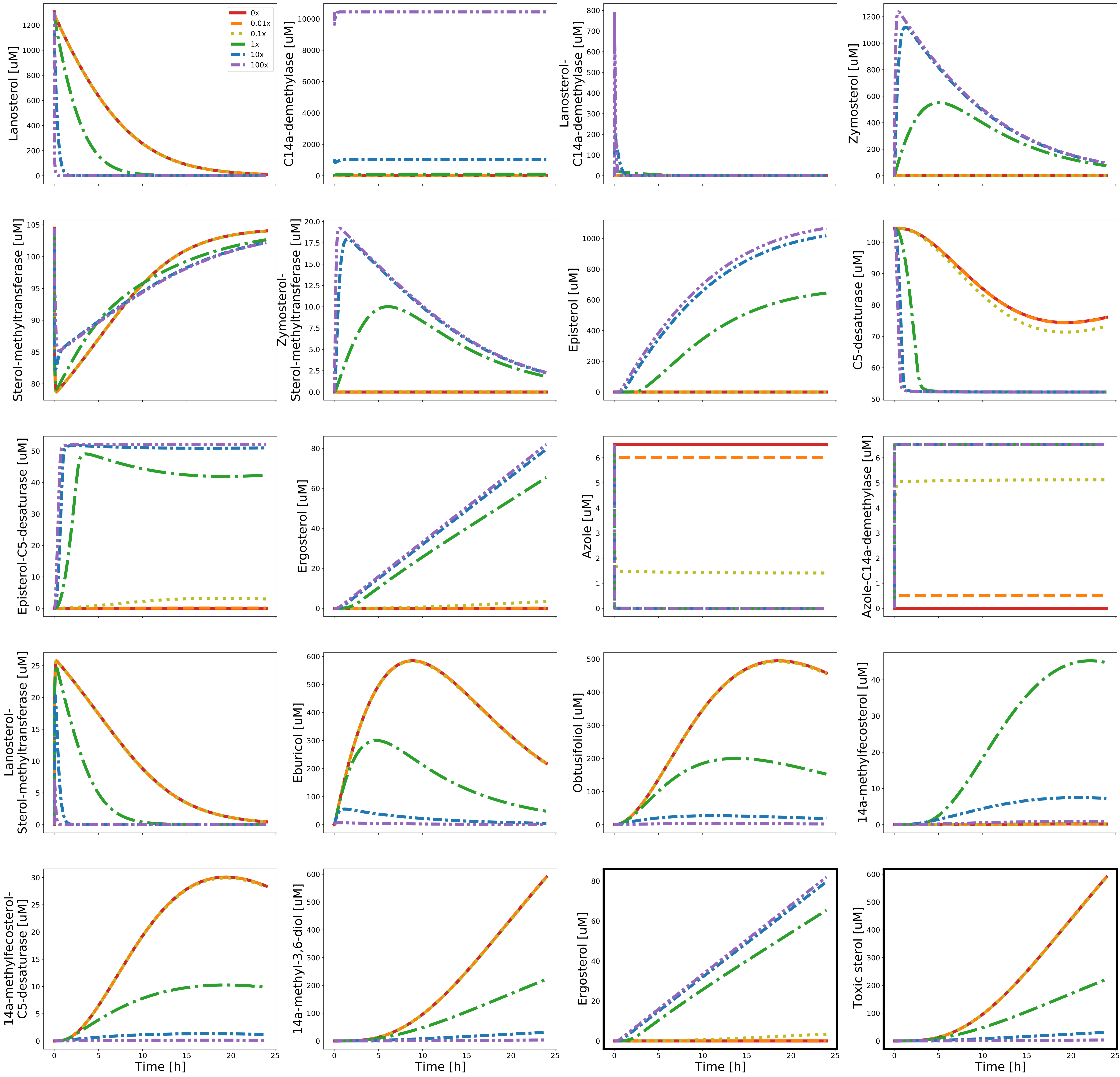

Supplement: FIG S3 [file msystems.00691-22-s0004.pdf]

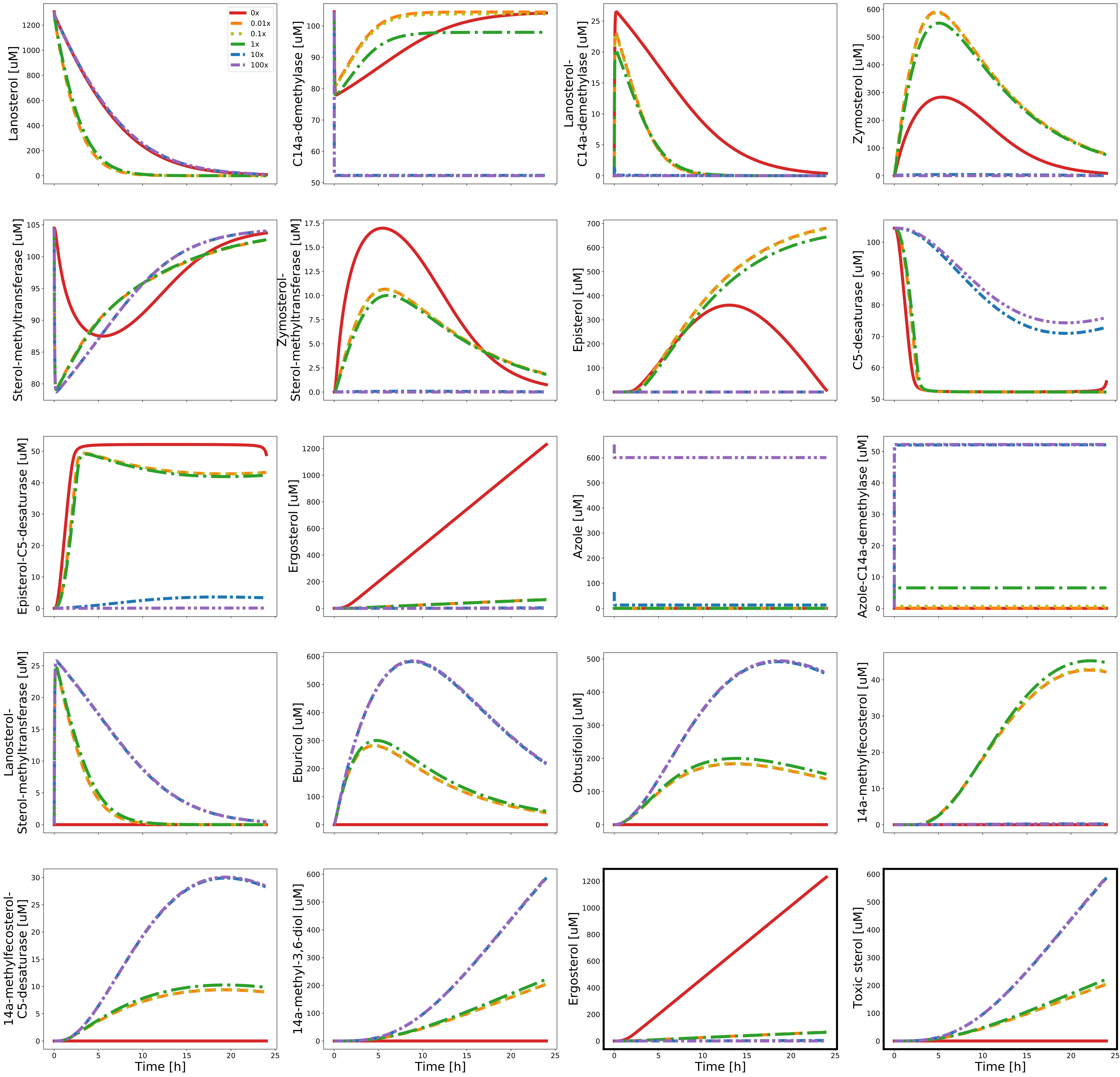

Supplement: FIG S4 [file msystems.00691-22-s0005.pdf]

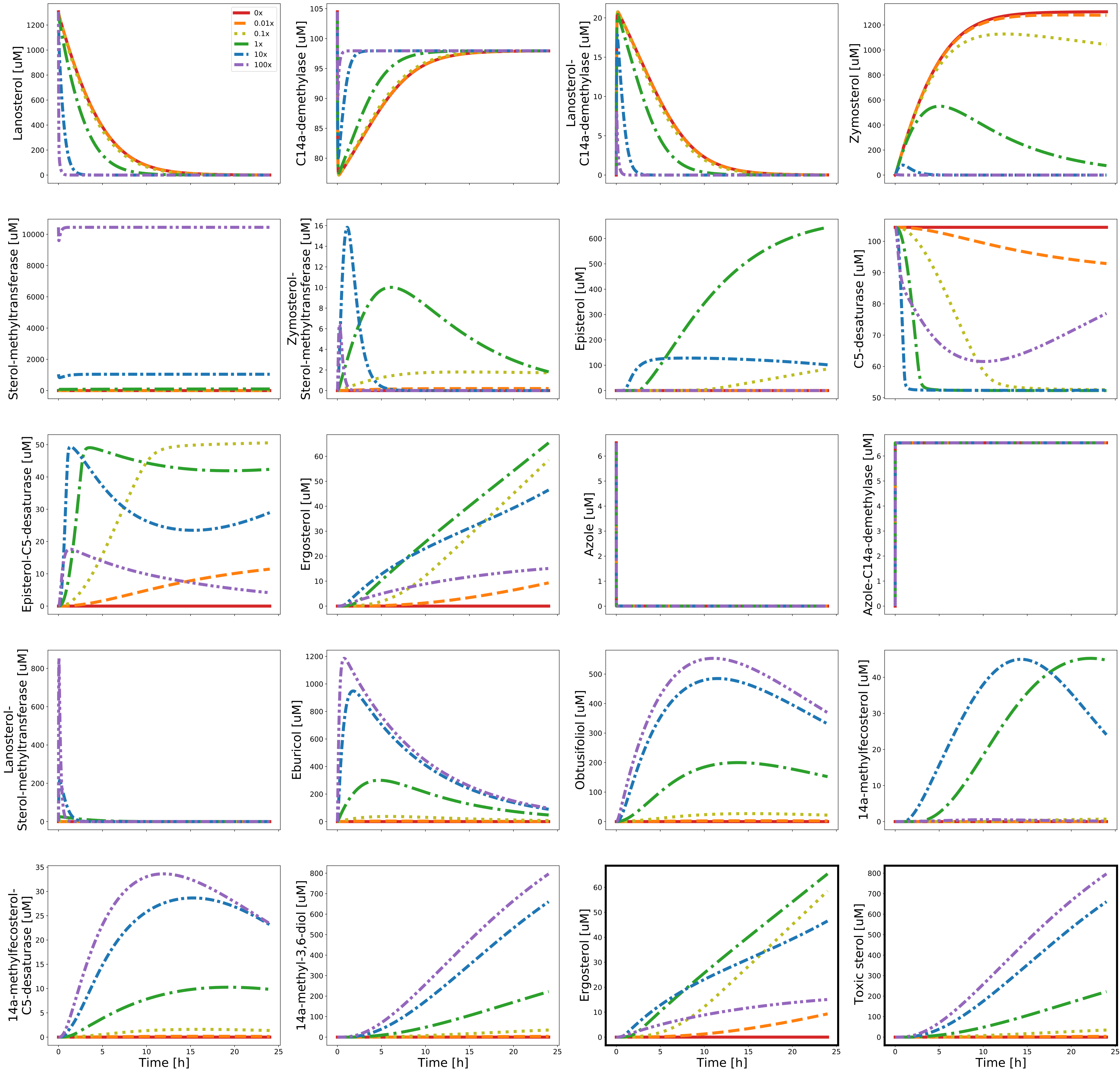

Supplement: FIG S5 [file msystems.00691-22-s0006.pdf]

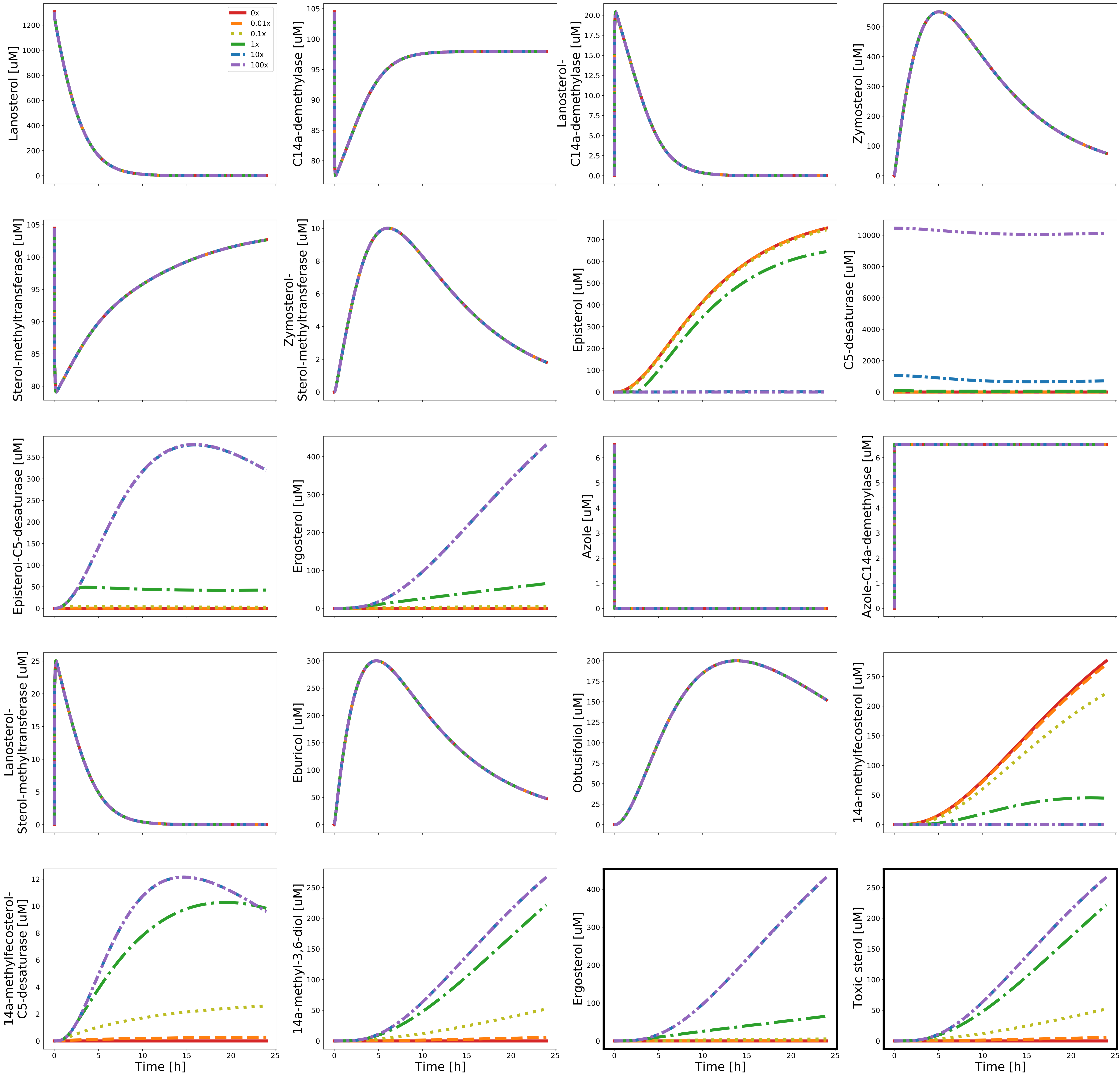

Supplement: FIG S6 [file msystems.00691-22-s0007.pdf]

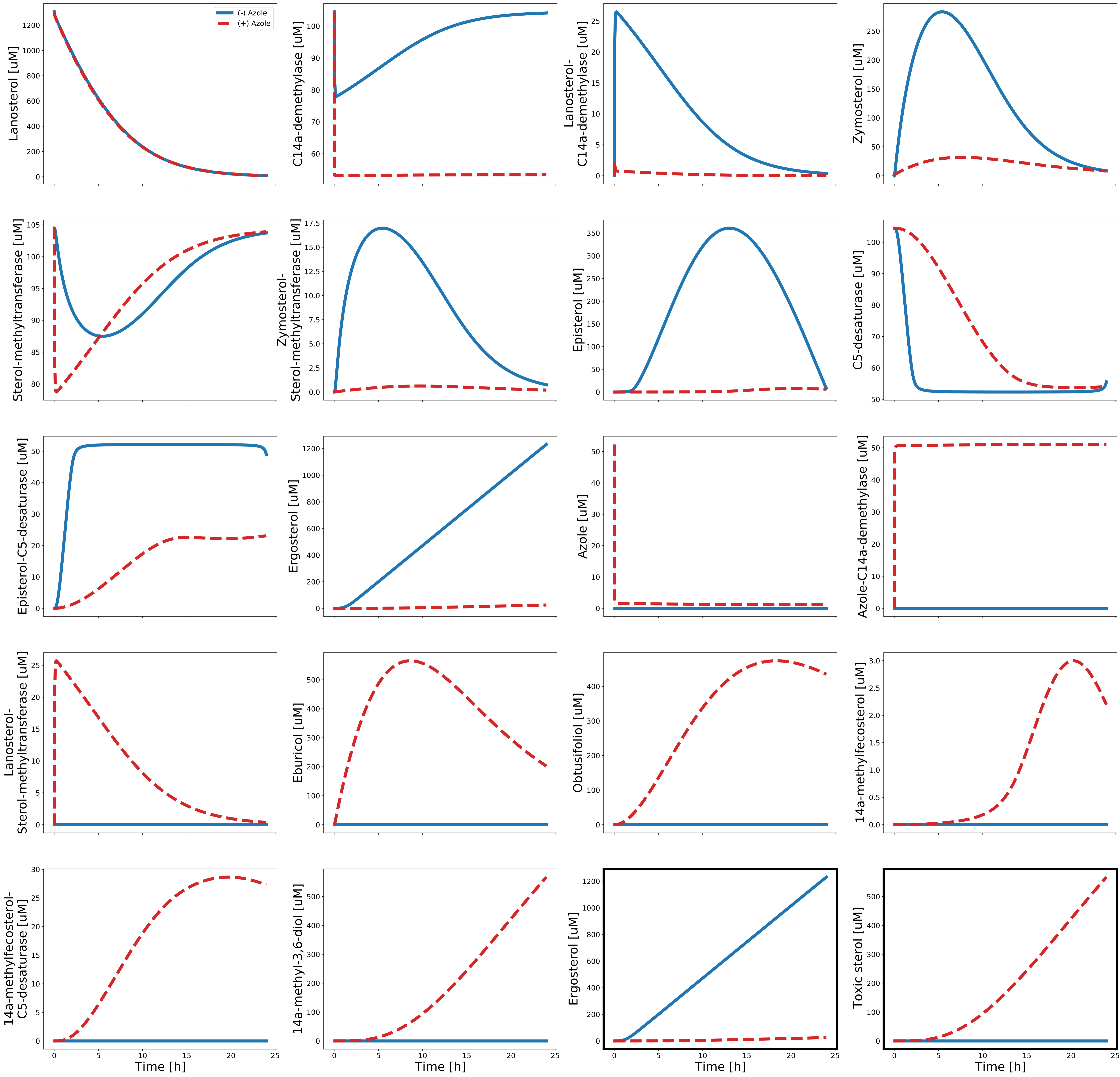

Supplement: FIG S1 [file msystems.00691-22-s0002.pdf]
