# Supplementary material for: Mathematical Modeling of Fluconazole Resistance in the Ergosterol Pathway of Candida albicans
Source: mSystems. 2022 Nov 16;7(6):e00691-22. doi: 10.1128/msystems.00691-22 (PMC9765018; doi:10.1128/msystems.00691-22)
Supplement: TABLE S1 [file msystems.00691-22-s0001.pdf]

| Notation | Description | Parameter Value | | Reference |
| --- | --- | --- | --- | --- |
| | | (-) Azole | (+) Azole | |
| $k_1$ | Forward rate constant for Lanosterol with C14α-demethylase | 0.0000825 $\mu M^{-1}$ $min^{-1}$ | 0.0001 $\mu M^{-1}$ $min^{-1}$ | Approximated from fitting to sterol composition of (1) |
| $k_{-1}$ | Backward rate constant for Lanosterol with C14α-demethylase | 0.1 $min^{-1}$ | 0.1 $min^{-1}$ | Approximated from fitting to sterol composition of (1) |
| $k_4$ | Catalytic production rate of C14α-demethylase | 0.1 $min^{-1}$ | 0.2 $min^{-1}$ | Approximated from fitting to sterol composition of (1) |
| $k_2$ | Forward rate constant for Zymosterol with Sterol-methyltransferase | 0.00017 $\mu M^{-1}$ $min^{-1}$ | 0.000045 $\mu M^{-1}$ $min^{-1}$ | Approximated from fitting to sterol composition of (1) |
| $k_{-2}$ | Backward rate constant for Zymosterol with Sterol-methyltransferase | 0.1 $min^{-1}$ | 0.1 $min^{-1}$ | Approximated from fitting to sterol composition of (1) |
| $k_5$ | Catalytic production rate of Sterol-methyltransferase (Episterol) | 0.1 $min^{-1}$ | 0.09 $min^{-1}$ | Approximated from fitting to sterol composition of (1) |
| $k_3$ | Forward rate constant for Episterol with C5-desaturase | 0.1 $\mu M^{-1}$ $min^{-1}$ | 0.1 $\mu M^{-1}$ $min^{-1}$ | Approximated from fitting to sterol composition of (1) |
| $k_{-3}$ | Backward rate constant for Episterol with C5-desaturase | 0.1 $min^{-1}$ | 0.1 $min^{-1}$ | Approximated from fitting to sterol composition of (1) |
| $k_6$ | Catalytic production rate of C5-desaturase (Ergosterol) | 0.017378 $min^{-1}$ | 0.001113 $min^{-1}$ | Approximated from fitting to sterol composition of (1) |
| $k_{1a}$ | Forward rate constant for Fluconazole with C14α-demethylase | 0 $\mu M^{-1}$ $min^{-1}$ | 0.1 $\mu M^{-1}$ $min^{-1}$ | Approximated from fitting to sterol composition of (1) |
| $k_{-1a}$ | Backward rate constant for Fluconazole with C14α-demethylase | 0 $min^{-1}$ | 0.0056 $min^{-1}$ | Calculated from $K_d$ of (2) |
| $k_{2a}$ | Forward rate constant for Lanosterol with Sterol- | 0 $\mu M^{-1}$ $min^{-1}$ | 0.000078 $\mu M^{-1}$ $min^{-1}$ | Approximated from fitting to sterol |

| | | | | |
|---|---|---|---|---|
| | methyltransferase | | | composition of (1) |
| $k_{-2a}$ | Backward rate constant for Lanosterol with Sterol-methyltransferase | 0 $min^{-1}$ | 0.1 $min^{-1}$ | Approximated from fitting to sterol composition of (1) |
| $k_{4a}$ | Catalytic production rate of Sterol-methyltransferase (Eburicol) | 0 $min^{-1}$ | 0.1 $min^{-1}$ | Approximated from fitting to sterol composition of (1) |
| $k_{3a}$ | Forward rate constant for 14α-methylfecosterol with C5-desaturase | 0 $\mu M^{-1}$ $min^{-1}$ | 0.4 $\mu M^{-1}$ $min^{-1}$ | Approximated from fitting to sterol composition of (1) |
| $k_{-3a}$ | Backward rate constant for 14α-methylfecosterol with C5-desaturase | 0 $min^{-1}$ | 0.1 $min^{-1}$ | Approximated from fitting to sterol composition of (1) |
| $k_{7a}$ | Catalytic production rate of C5-desaturase (14α-methyl-3,6-diol) | 0 $min^{-1}$ | 0.02134 $min^{-1}$ | Approximated from fitting to sterol composition of (1) |
| $k_{5a}$ | Forward rate constant for Eburicol to Obtusifoliol | 0 $\mu M^{-1}$ $min^{-1}$ | 0.00181 $\mu M^{-1}$ $min^{-1}$ | Approximated from fitting to sterol composition of (1) |
| $k_{6a}$ | Forward rate constant for Obtusifoliol to 14α-methylfecosterol | 0 $\mu M^{-1}$ $min^{-1}$ | 0.0013 $\mu M^{-1}$ $min^{-1}$ | Approximated from fitting to sterol composition of (1) |

## References


1. Kelly SL, Lamb DC, Kelly DE, Manning NJ, Loeffler J, Hebart H, Schumacher U, Einsele H. 1997. Resistance to fluconazole and cross-resistance to amphotericin B in *Candida albicans* from AIDS patients caused by defective sterol Δ5,6-desaturation. FEBS Lett 400:80–2.

2. Warrilow AG, Parker JE, Kelly DE, Kelly SL, 2013. Azole affinity of sterol 14α-demethylase (*CYP51*) enzymes from *Candida albicans* and *Homo sapiens*. Antimicrob Agents Chemother 57:1352-1360.
